# Pediatric Hospital Care Research Priorities Within a Regional Whole‐of‐Life Health Service: Established Through Consumer and Healthcare Professional Collaboration

**DOI:** 10.1155/ijpe/7312620

**Published:** 2026-08-28

**Authors:** Julie Creen, Breanna Medcalfe, Annelise Jefferies, Clare Thomas, Roni Cole

**Affiliations:** ^1^ Women′s and Children′s Services, Sunshine Coast Hospital and Health Service, Birtinya, Queensland, Australia; ^2^ Maternal and Child Health, Sunshine Coast Health Institute, Birtinya, Queensland, Australia; ^3^ Consumer Partner for the Paediatric Department, Sunshine Coast Hospital and Health Service, Birtinya, Queensland, Australia; ^4^ Clinical Excellence Queensland, Queensland Health, Brisbane, Australia, health.qld.gov.au

**Keywords:** consumer, decision-making, James Lind Alliance, pediatric hospital care, priority setting, regional health service, research agenda, research prioritization, whole-of-life health service

## Abstract

**Aim:**

This study is aimed at establishing the top research priorities in pediatric hospital care that are relevant and meaningful to local pediatric consumers and healthcare professionals to optimize local service outcomes.

**Methods:**

This study adopted a modified consensus‐based research priority‐setting design, utilizing James Lind Alliance (JLA) methodology led by a steering committee comprising consumers (parents, carers, and previous patients) and healthcare professionals. The study was conducted at a regional Australian tertiary hospital providing whole‐of‐life health services from April 2023 to June 2024. The six‐stage study design consisted of (1) initiation, (2) consultation, (3) collation, (4) interim ranking, (5) final priority setting, and (6) dissemination.

**Results:**

Equal representation of consumers and healthcare professionals was achieved during the priority‐setting processes, with active consumer partnership during research design, data collection, analysis, and dissemination phases. A total of 104 unique research submissions were generated through the harvesting survey (*n* = 104), and following screening and synthesis, 21 indicative research uncertainties were identified. Following interim prioritization (*n* = 110) and final refinement through a consensus workshop (*n* = 24), the Top 5 pediatric research priorities were established: reducing healthcare‐related trauma, upscaling pediatric‐centered support strategies and infrastructure, enhancing effective communication, enhancing adolescent and young adult services, and improving efficiency and care as children move through a whole‐of‐life health service.

**Conclusion:**

These research priorities align with those of other international pediatric healthcare services while highlighting unique local contextual differences. Notably, reducing trauma associated with healthcare experiences emerged as a central priority. Our modified JLA approach appears to be an effective framework for engaging research end‐users in establishing relevant and meaningful research priorities tailored to local health‐service needs. Future initiatives should continue to ensure consumers are active coinvestigators to ensure outcomes remain patient‐centered and community‐focused.

## 1. Introduction

With demand for healthcare services exceeding supply, the need for high‐quality evidence‐informed public health services is greater than ever. The Australian National Clinical Trials Governance Framework outlines systems and processes that health service organizations should establish, aligning with the World Health Organization and the United Nations Convention for the Rights of the Child, which consistently emphasize the need for accessible, relevant healthcare that includes the voice and involvement of consumers, particularly children, families, and caregivers within pediatric settings [1–4].

Collaboratively developing research agendas with consumers and healthcare professionals (HCPs) enhances translational impact on health service delivery and ensures findings are directly applicable, optimizing patient outcomes and experiences [4, 5]. Meaningful consumer engagement places their needs at the center of research, improving research relevance and the efficient use of finite resources [5, 6].

Research priority‐setting processes that exclude consumer involvement often result in condition‐specific or biomedical research agendas, lacking alignment with questions most important to patients and families, limiting applicability to broader pediatric care settings [6, 7]. A recent scoping review by Modanloo et al. [8] identified only 30 studies that included children, youth, or their families in healthcare research priority‐setting processes. Furthermore, studies that have involved consumer engagement are largely condition‐specific, limiting generalizability to peripheral generalist pediatric hospital caseloads [8, 9]. Canada has identified the Top 10 research questions for general pediatric hospital care [10], and some recent studies in Australia have applied a consumer and healthcare‐driven approach for identifying research priorities for community‐ and hospital‐based pediatric caseloads [11–13].

The James Lind Alliance (JLA) method is a staged process that ensures equal participation of consumers and HCPs in prioritizing future research pertinent to patient experience and clinical outcomes [14]. Partnering equally with these stakeholders in research agenda setting has demonstrated benefits at multiple levels: improved patient outcomes, enhanced clinician engagement and job satisfaction, and societal benefit with improved systems of care [15, 16]. This study was aimed at establishing the top research priorities for pediatric hospital care in regional Australia that are relevant and meaningful to local consumers and HCPs. The objectives were to (1) identify and prioritize unanswered questions from children, families, and pediatric HCPs about hospital care for children and young people and (2) achieve a collaborative consensus on the top pediatric hospital care research priorities for the local pediatric department.

## 2. Methods

### 2.1. Study Design

The study used a consensus‐based research priority‐setting approach, adopting a modified JLA methodology. The project followed the six core stages described in the JLA guidebook, including establishment of a steering committee, uncertainty gathering, uncertainty collation, interim prioritization, final consensus workshop, and dissemination. However, several modifications were made. First, a formal JLA priority‐setting partnership was not established, and an accredited JLA adviser was not engaged. Second, evidence checking was limited to searches of PubMed for systematic reviews rather than comprehensive multidatabase evidence verification. Third, the final workshop was facilitated internally by independent facilitators rather than JLA‐appointed advisers. These modifications were made due to resource constraints and the focus of the local project. Despite these adaptations, the study maintained the core JLA principles of equal consumer–clinician partnership, transparent decision‐making, and consensus‐derived priority setting. This modified JLA methodology is a proven approach that has been successfully utilized in other studies [11–13, 17, 18]. This methodology was adopted to ensure equitable integration of consumer lived experience and HCP expertise.

Ethical approval for this project was granted by the Metro North B Human Research Ethics Committee (HREC/2023/MNHB/97144). Our reporting aligns with the Reporting Guideline for Priority Setting of Health Research (REPRISE) [19].

### 2.2. Setting and Scope

This study was conducted between April 2023 and June 2024 at a public, regional Australian tertiary teaching facility offering whole‐of‐life services. Establishing research priorities was essential following a recent service expansion to focus finite research resources on addressing local evidence‐practice gaps with clinical and societal relevance. In this study, relevance was defined as research uncertainties identified by our health service′s pediatric community, including children, families, and HCP, through the structured priority‐setting process.

Participants included two key stakeholder groups: (a) consumers, defined as individuals with lived experience of the pediatric health service, including parents, carers, and young adults 18 years and older who were currently accessing pediatric services or who had accessed services in the past 3 years, and (b) pediatric HCP. Participation in this study was voluntary and based on informed consent. Prior to participation, all individuals received written information describing the purpose of the study, the nature of participation, expected time commitment, potential risks and benefits, data handling procedures, and the voluntary nature of involvement. Participants were informed that they could decline to answer any question and could withdraw from the study at any time without consequence. Consent was implied through voluntary completion and submission of study surveys. Written informed consent was obtained from participants involved in priority‐setting workshops and research meetings.

Aligned with the JLA process, the project steering committee defined a clear scope. Research suggestions were considered in scope if they were related to healthcare, treatment, or management within the inpatient or outpatient pediatric services and deemed feasible for the department. Mental health and neonatal healthcare topics were included if they related to services delivered by the pediatric department.

### 2.3. Priority‐Setting Process

**Figure 1:**
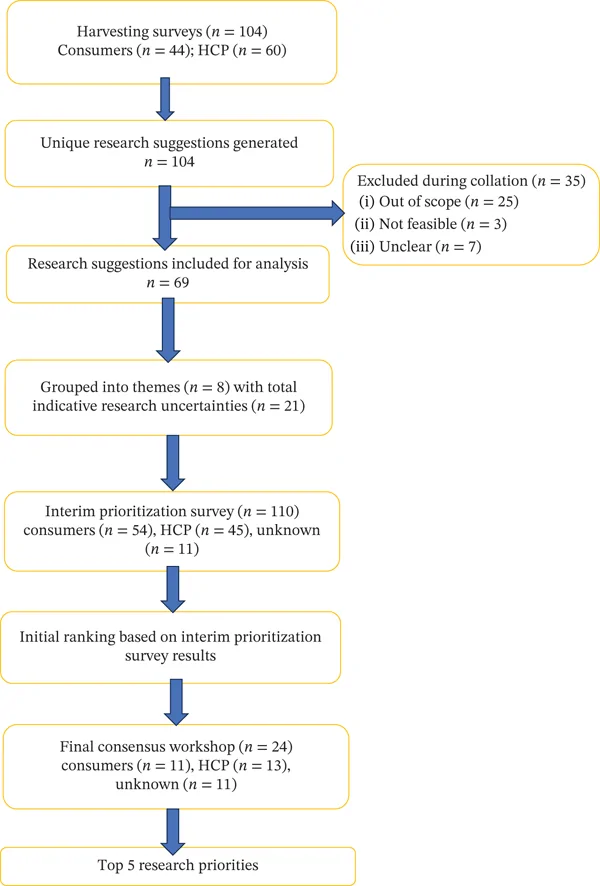
Overview of priority‐setting results.

The study employed a six‐stage study design consisting of (1) initiation, (2) consultation (harvesting survey), (3) collation and verification of uncertainties, (4) interim prioritization survey, (5) final priority setting, and (6) dissemination (see Figure 1).

#### 2.3.1. Stage 1: Initiation

The project was overseen by a steering committee of representatives from key stakeholder groups consisting of HCP (*n* = 6) including two pediatricians, two allied health practitioners, a nurse, and a nurse–researcher and consumers (*n* = 4) with lived experience, including three parents of children who had previously or were currently accessing pediatric hospital care, and a young adult with prior lived pediatric hospital care experience. Committee members were recruited in April 2023 through convenience and purposive sampling, ensuring diversity in ethnic, cultural, and clinical experiences. Terms of reference were developed to outline the committee′s purpose, roles, responsibilities, and decision‐making processes. Meetings were held both in person and via video conference. To ensure meaningful and authentic engagement, HCP were provided with paid nonclinical time to participate, and consumers were offered remuneration, including paid parking.

The steering committee codesigned the study, defined the scope, guided survey development, and oversaw all stages of data analysis to ensure fidelity to stakeholder perspectives. Steering member consumers′ input helped shape engagement strategies to optimize inclusivity and accessibility at all stages, ensuring all participants could provide valuable, informed feedback.

#### 2.3.2. Stage 2: Consultation (Harvesting Survey)

The cross‐sectional harvesting survey was cocreated by the steering committee to gather stakeholder perspectives related to pediatric hospital care research needs (see Supporting Information 1: File S1). Survey development was modeled on previous JLA examples and piloted by the steering committee (*n* = 10; consumers *n* = 4, HCP *n* = 6) to ensure clarity, readability, and acceptability. Iterative refinements were made to ensure questions were meaningful to both consumers and clinicians. The survey was hosted on Qualtrics and disseminated electronically via QR code between September 2023 and December 2023. HCPs were recruited in‐person, via managerial email networks, posters, and snowball sampling. Consumers were recruited in‐person via invitation to families in the clinic waiting rooms or in‐patient unit, posters, and hospital social media platforms. There was no limit placed on the number of suggestions participants could submit. Survey closure was time‐based (3 months) and aligned with JLA guidance.

#### 2.3.3. Stage 3: Collation and Verification of Uncertainties

Using a structured qualitative content analysis approach, the research team compiled survey responses using the JLA templates outlined in the guidebook [14]. Two researchers, A.J. and R.C., independently coded submissions and grouped them into primary categories through inductive thematic analysis. Where possible, original wording was preserved; however, phrasing was refined to ensure clarity and avoid duplication. Researchers J.C. and C.T. independently verified categorization. The steering group exercised oversight to ensure raw data were interpreted appropriately during data analysis and collation. Similar suggestions within categories were merged and phrasing refined to form a single indicative summary of research uncertainties. Differences were resolved through discussion and review by the steering committee until consensus was achieved and all final uncertainties were endorsed.

Research uncertainties were checked by the steering committee against existing sources of evidence. PubMed was searched in January 2024 using combinations of terms derived from each indicative research uncertainty together with filters for systematic reviews and meta‐analyses. Research uncertainty was retained if no recent high‐quality systematic review addressing the question was identified or if existing evidence remained inconclusive.

#### 2.3.4. Stage 4: Interim Prioritization Survey

An interim ranking online survey of the research uncertainties was redistributed back to the pediatric hospital community (following the same networks and processes used for the initial harvesting survey during Stage 2). The interim prioritization survey was available online via Qualtrics between February and March 2024. Participants were asked to select up to 10 research uncertainties and then rate each selected topic on a sliding scale from 0 to 100 to indicate their perceived importance of the uncertainty (0 being *not important*).

Analysis followed JLA guidance [14] and was conducted in Excel, where rankings were based on frequency of selection and aggregated importance scores. Results were compiled and stratified by consumer and HCP responses, providing individual stakeholder groups and total combined rank‐ordered lists according to the total vote count. A “rate of importance” score was derived by averaging the importance ratings for each research uncertainty. Participants were included in the analysis if they had responded to all interim ranking items. If demographic questions were left unanswered, these data were recorded as unknown, but the participant′s ranking data responses were retained.

#### 2.3.5. Stage 5: Final Priority‐Setting Workshop

**Figure 2:**
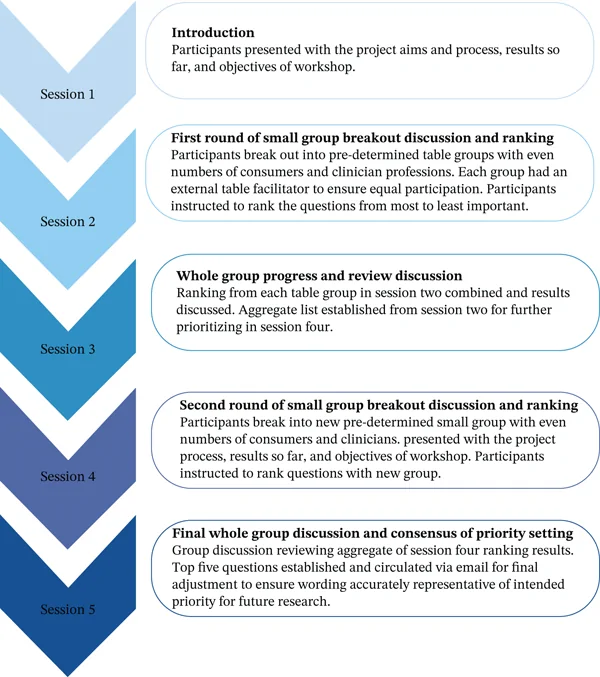
Modified nominal group technique workshop priority‐setting process.

A purposive sample of consumers and HCPs was recruited from survey participants and in‐person invitations to participate in the final priority‐setting workshop. Purposive sampling ensured a broad representation of key stakeholder groups. Participation involved a 6‐h face‐to‐face workshop in March 2024 using the modified nominal group technique established in the JLA guidebook (see Figure 2). All consumers were provided with remuneration for their time and parking expenses to attend the workshop. Independent facilitators guided and moderated two sessions of five small breakout groups where all research uncertainties were reviewed and ranked into green (most important), yellow, and red zones. Iterative rounds of discussion and reranking were conducted, and then rankings were consolidated across groups. At the conclusion of each breakout session, small groups needed to have all uncertainties ranked from most important to least important. Small‐group composition was balanced between stakeholder group representatives (i.e., equal balance of consumers and healthcare profession disciplines), with the facilitators ensuring each participant in the breakout group had an opportunity to contribute to discussion and ranking decisions.

Analysis was conducted in Excel with an aggregate ranking determined. Finally, through structured group discussion, participants reached consensus on the top research priorities. Consensus was defined as agreement among workshop participants following facilitated discussion, with no substantial objections remaining after final ranking.

#### 2.3.6. Stage 6: Dissemination

The final top research priorities were disseminated through infographics, reports, local symposiums, and national conference presentations. Key priorities have been embedded into the department′s research strategy and will guide future research planning, grant development, and collaborations.

### 2.4. Patient and Public Involvement

Consumer representatives were actively involved throughout the priority‐setting process, from developing the study design as members of the steering committee, as participants in the survey and workshop, and in the process of analysis, reporting and dissemination. Table 1 provides reflective quotes from consumer steering committee members, offering additional details regarding their involvement and engagement across the research process. These reflections highlight the meaningful consumer integration achieved during this priority setting, including enhanced public trust, satisfaction, and credibility of the research activity.

**Table 1:** Consumer participatory researcher and engagement quotes.

Research stage	Quotes
Stage 1: Initiation (steering committee)	“The steering committee and fellow authors provided support to ensure that myself and other consumers involved always had a valuable voice at the table and were meaningfully consulted throughout the whole process. I felt this added significant value to the research method and results, as it had genuine consumer involvement every step of the way.”
	“I was able to provide advice and experiences from the specific hospital and health service and also draw in knowledge from other areas. I had recent experience as a long‐term patient with a disability in the pediatric sector, which meant I was able to provide insight as someone who had real‐life experience.”
Stage 2: Consultation (harvesting survey)	“In the formulation of the surveys, I felt that my feedback as a consumer ensured the surveys were easily understood and would be well received by consumers.”
	“Consumer feedback was particularly crucial in the successful engagement and dispersion of the surveys, as an identified challenge was how to obtain a wide reach and diversity in survey participants. The consumer representatives brainstormed options together with the steering committee on how to best connect with this demographic to achieve quality and diverse research results.”
Stage 3: Collation of survey results	“This was a great opportunity for me as a consumer participatory researcher to provide unique and patient‐focused feedback toward the theming of results to inform the next stage of the process.”
Stage 4: Interim prioritization	“Again, consumer feedback during participation in the steering committee directly informed the structure and content of the interim ranking survey, ensuring that it was accessible and easily read and understood by all participants, especially other consumers.”
	“Opening the survey to such a wide variety of participants showed this study′s commitment to obtaining data from a diverse population to ensure that the final results were inclusive of the needs of our hospital and health service (HHS).”
Stage 5: Final priority setting	“Wider consumer and patient engagement were sought during the workshop phase, and equal representation between clinicians and consumers was evident, ensuring the final priority setting of the research questions was representative of the hospital and wider community. This involvement of consumers as members and representatives of the wider community ensured the prioritized research questions moving forward were an accurate representation of how the health service can best serve the community in this region.”

## 3. Results

A total of 104 participants contributed to the harvesting survey, generating 104 research submissions. Following screening and synthesis, 21 indicative research uncertainties were identified and progressed to interim prioritization (*n* = 110 participants). These were subsequently refined through a final consensus workshop involving 24 participants, resulting in the Top 5 research priorities for pediatric hospital care. Figure 1 presents a summary flowchart of the main results across the study process. Table 2 provides participant demographics for each priority‐setting stage.

**Table 2:** Participant characteristics at each stage of the priority‐setting process.

Characteristic	Harvesting survey (*n* = 104) (%)	Interim ranking survey (*n* = 110) (%)	Final workshop (*n* = 24) (%)
*Stakeholder representation*			
Consumer	44 (42.3)	54 (49.1)	11 (45.8)
Clinician	60 (57.7)	45 (40.9)	13 (54.2)
Unknown	0 (0.0)	11 (10.0)	0 (0.0)
*Consumer representation*	*n* = 44	*n* = 54	*n* = 11
Current consumer	34 (77.3)	40 (74.1)	4 (36.4)
Consumer in last 3 years—Inclusive of consumer advocates	10 (22.7)	14 (25.9)	7 (63.6)
*Clinician*	*n* = 60	*n* = 45	*n* = 13
Medical officer	8 (13.3)	12 (26.7)	4 (30.8)
Nurse	13 (21.7)	11 (24.4)	3 (23.1)
Allied health	13 (21.7)	19 (42.2)	6 (46.1)
Unknown	26 (43.3)	3 (6.7)	0 (0.0)
*Aboriginal and/or Torres Strait Islander identity*	1 (1.0)	3 (2.7)	1 (4.2)
*Gender*			
Female	54 (51.9)	45 (40.9)	24 (100.0)
Male	5 (4.8)	5 (4.5)	0 (0.0)
Prefer not to say	0 (0.0)	3 (2.7)	0 (0.0)
Unknown	45 (43.3)	57 (51.8)	0 (0.0)

### 3.1. Harvesting Survey and Verification of Uncertainties

A total of 104 participants comprising consumers (*n* = 44) (42%) and HCPs (*n* = 60) (58%) responded to the harvesting survey, coincidently with a total of 104 unique research suggestions generated once duplicates were removed. A total of 35 responses (34%) were excluded due to being out‐of‐scope (*n* = 25), infeasible for local research (*n* = 3), or unclear suggestions (*n* = 7). The remaining (69, 66%) suggestions were grouped into eight primary categories: neurodevelopmental and neuroaffirming (*n* = 6), feeding and nutrition (*n* = 7), barriers to discharge (*n* = 5), models of care and service delivery (*n* = 25), respiratory and medical (*n* = 4), consumer experience and engagement (*n* = 10), procedures and treatments (*n* = 9), and miscellaneous (*n* = 3). Within each category, similar submissions were merged to form 21 indicative research uncertainties, ensuring fidelity to participants′ original suggestions. These were verified as unanswered research uncertainties. Survey abandonment was minimal for primary question submission but was observed in demographic fields.

### 3.2. Interim Prioritization

The 21 research uncertainties were distributed via an interim prioritization survey, completed by 110 participants (consumers *n* = 54, 49.1%; HCP *n* = 45, 40.9%; unknown *n* = 11, 10.0%). There was no survey abandonment across ranking questions, with 10% abandonment in demographic questions at the end resulting in unknown participant demographics. Table 3 provides detailed interim prioritization rankings of the 21 research uncertainties with importance rated by frequency of selection and mean importance scores. The highest‐ranked uncertainty was consistent across both stakeholder groups (consumers and HCPs), being “reducing healthcare‐related trauma and traumatic interactions for pediatric patients.” Themes relating to models of care, communication, and transitions between services also ranked within the Top 5 for both stakeholder groups.

**Table 3:** Interim prioritization research topic ranking and rate of importance scores.

Research topic	Total count (*n* = 110)	Overall ranking	Clinician ranking	Consumer ranking	Rate of importance^a^
Reducing healthcare‐related trauma and traumatic interactions for pediatric patients, including support for clinical procedures and general hospital care	70	**1**	**1**	**1**	83
Efficiency and quality of care moving children through the hospital healthcare system	60	**2**	**2**	**3**	72
Models of care that support a young person′s needs and well‐being as they move from children′s services to adult services	59	**3**	**5**	**2**	78
Defining effective communication and the impact of this on children and family experience, including consistent messaging and standardized information giving	55	**4**	**4**	**5**	79
Impact of clinical care on sleep disruption during hospital admission	52	**5**	12	**5**	73
Barriers and enablers to discharge into the community for ongoing care	51	**6**	**2**	**10**	65
Strategies to deliver continuity of care between and across treating teams	49	**7**	16	**3**	76
Implementation and evaluation of patient‐centered support strategies and infrastructure for families during hospital care, for example, children′s preferences, parental amenities, parental meal support, games and play, mental well‐being, and breastfeeding	48	**8**	**10**	**8**	76
Nutritional support for pediatric patients	47	**9**	15	**5**	72
Impact on neurodevelopment of children exposed to illicit substances and alcohol in utero	46	**10**	**5**	13	72
Models of care for medical admissions of children/youth with eating disorders	46	**10**	**5**	12	80
Barriers and enablers to engage staff in ongoing education	45	12	12	**10**	79
Best in‐patient care for children and young people with respiratory illnesses	44	13	**9**	17	78
Insertion and management of intravenous (iv) devices for children	41	14	12	13	78
Impact of premature birth and early intervention on children′s learning and executive functioning skills	40	15	**10**	17	74
Processes and communication for effective interhospital (between hospitals) and intrahospital (within the hospital, for example, between emergency and the ward) transfers of children/youth	40	15	18	**9**	72
Impact of hospital in the home (HiTH) models of care and family experience	39	17	**5**	19	69
Health benefits to blended real food feeds over formula	32	18	19	16	62
Methods of monitoring treatment modalities and outcomes on Type 1 diabetes, including devices, lifestyle intervention, and patient experience	28	19	20	13	85
Impact of early intervention on fine motor development for infants at risk of cerebral palsy	27	20	16	21	74
Group education for family diabetes care	20	21	21	20	77

*Note:* Bolded numbers indicate stakeholder representative group Top 10 ranked research topics.

^a^Rate: 0 = *least important* and 100 = *most important*.

### 3.3. Workshop Outcomes

Twenty‐four participants (consumers *n* = 11; HCP *n* = 13) attended the final prioritization workshop. HCPs included medical (*n* = 4), nursing (*n* = 3), and allied health (*n* = 6), including social work, pharmacy, physiotherapy, dietetics, and occupational therapy (Table 2). The 21 research uncertainties were reviewed and ranked through two rounds of facilitated small‐group discussions using a modified nominal group technique. Consensus was achieved on the final outcomes, which detailed the Top 5 priority questions with associated subtopics (Table 4). Notably, the topic of reducing healthcare‐related trauma consistently ranked as the top priority for both consumers and HCPs throughout both the interim prioritization and final workshop stages.

**Table 4:** Top 5 research priorities.

Research priorities
**Priority 1: What are ways to reduce healthcare-related trauma and traumatic interactions for pediatric patients and their families?**
Strategies to improve support during clinical interventions such as nasogastric insertions, diagnostic procedures, and needle procedures
Establish and evaluate psychological and physical safe spaces to access and receive pediatric healthcare
**Priority 2: How do we better provide pediatric-centered support strategies and infrastructure for children and their families during hospital admission?**
Investigate sibling and parental needs to optimize the delivery of holistic care to children/young people and their families, such as nutrition, sleep, and mental health
Improve healthcare experiences and outcomes through incorporating children and adolescents′ preferences and needs
**Priority 3: What are strategies to enhance effective communication to improve the child/adolescent and family′s experience and outcomes?**
Improve consistency and clarity of the information children/adolescents and their families receive, such as standardized information sharing
Optimize continuity of care between and across treating teams
Upscale processes for effective inter‐ and intrahospital pediatric transfers
**Priority 4: How do we enhance adolescents′ and young adults′ engagement, experiences, and outcomes as they move from pediatric services to adult services?**
Innovative adolescent and young adult models of care
Address factors that build or erode trust and engagement in adolescent and young adults′ healthcare
**Priority 5: How do we improve the efficiency and quality of care as children move through a tertiary level “whole-of-life” hospital?**
Optimize tertiary pediatric healthcare within alternative delivery settings
Investigate facilitators and barriers to inpatient and outpatient discharge
Enhance patient safety and upscale innovative models of care

## 4. Discussion

This priority‐setting project effectively engaged HCPs alongside pediatric department consumers to establish the top priorities for general pediatric hospital care research, utilizing a modified JLA approach. This method successfully identified research priorities aligned with the values and needs of the primary research end‐users—children, families, carers, and the HCPs who work with them.

Through employing a conceptual approach, this study positions consumers and HCPs at the forefront and moves beyond tokenistic involvement. This aligns with the current literature that underscores the benefits of meaningful and authentic consumer engagement throughout the entire research process to ensure relevance and applicability of findings [4, 8, 12, 13]. However, despite documented benefits, the practical challenges researchers face in partnering with consumers often impact the successful implementation of ideal consumer participatory principles [11, 20]. The meaningful, sustained, and authentic inclusion of consumers through all phases of this project has demonstrated that the modified JLA process is an effective framework for consumer engagement when setting health service research agendas, as seen in other emerging studies adopting this approach in pediatrics [11–13].

Aligned with best practice consumer participatory research principles [4, 21], this study actively sought equitable representation of both consumers and HCPs throughout all stages of the research process. This commitment was established from the outset with a balanced steering committee of four consumers and six HCPs who collaboratively contributed to design choice and protocol. To continually facilitate equitable consumer engagement, the study implemented measures such as providing financial remuneration, offering both online and in‐person engagement platforms, and accommodating flexible scheduling to suit the needs of consumers and HCPs. While equitable representation from consumers and HCPs was achieved overall, HCPs were more responsive in the harvesting survey. Barriers to engagement for children and families are likely attributed to limited confidence, awareness, and experience in participating in research, as well as challenges in articulating complex healthcare needs and being confident to express their perspectives in the research process [8, 22].

An important challenge in this study was the limited direct involvement of children, due to ethical, societal, and logistical constraints necessitating reliance on adult proxies for most stages, as also highlighted in the literature [23]. Children′s rights to participate in decision‐making and access healthcare are well established [3, 11], and capturing their voices is imperative to understanding their unique perspective and priorities as primary consumers of pediatric healthcare [3, 11, 24]. This study prioritized efforts to include a young adult consumer within the steering committee who had recent long‐term pediatric healthcare experience; however, no children participated in the steering committee. Future research should further explore effective methods for engaging and partnering with children as active contributors throughout the research process.

Similar to research conducted in pediatric healthcare in Western Australia [12, 13], this project contributes to the emerging evidence of using the JLA priority‐setting approach within a regional general pediatric setting in a whole‐of‐life healthcare service in Australia. In contrast to many large‐scale or specialist‐specific JLA studies [10, 11, 25], this study successfully tailored and implemented the JLA process for a regional health service, demonstrating its adaptability and relevance for a local context. This approach yielded highly relevant findings for the regional healthcare organization, healthcare providers, and the children and families they serve.

As research demands on healthcare organizations increase amid limited resources, this study represents an important step toward establishing a robust research agenda that resonates with the clinical priorities of the pediatric interprofessional healthcare providers. By identifying strategic research priorities, this approach can help focus finite resources, strengthen healthcare organization competitiveness for research funding, and support the translation of evidence into practice.

The five research priorities identified align with those from other pediatric healthcare services [10–12] while also reflecting some localized contextualized differences. Similarities exist with international studies conducted in Canada where important research priorities for pediatric hospital care involve the child and family/caregivers′ experience and inclusion in decision‐making processes through effective communication, shared care, understanding of healthcare models, and support for transition through healthcare services [10–13]. Reducing the impact of trauma, including medical trauma, procedural support, pain management, and implementation of trauma‐informed care, has been identified in other studies [8, 10, 11], and locally it emerged as the highest priority, underscoring a unique emphasis among participants. It is acknowledged that these studies focused on disease‐specific populations, in contrast to this study, which focused on regional generalized pediatric care.

Notably, when HCPs and families are the primary drivers in establishing a research agenda, the focus tends to shift toward child and family experience and long‐term well‐being, rather than specific clinical biomedical questions. While evidence‐based practice for clinical outcomes is imperative, the manner in which care is delivered, including how the child and family are engaged, is critical. An example from this study is the priority placed on adolescent and young adult models of care, which did not emerge in other consumer‐driven research studies however aligns with Australian and State healthcare policy priorities [26, 27]. This local relevance highlights the importance and feasibility of exploring research priorities within a regional healthcare service, adding valuable knowledge to the global healthcare workforce on such matters. Given resource constraints in regional centers, research focused on relevant healthcare delivery and experiential priorities holds significant value for regional services, supporting impactful, practice‐oriented research that can be realistically implemented.

### 4.1. Strengths and Limitations

A key strength of this study was the inclusion of a diverse range of stakeholders, including parents, carers, and young adults with current or recent lived experience of the pediatric health service, alongside representation from multiple pediatric HCP disciplines. The adoption of a modified JLA methodology provided a structured and systematic approach to identifying research priorities for pediatric hospital care in regional Australian healthcare settings. This approach enabled varied perspectives to be considered and increased the likelihood that the resulting priorities reflect the needs of a whole‐of‐life regional pediatric service and are meaningful to those who use and deliver care.

While the authors sought comprehensive representation with a focus on consumer and HCP diversity, socioeconomic and cultural demographics were not recorded and therefore restrict insights into the diversity of backgrounds represented. Involvement from children and young people was minimal, and these absent voices must be considered a limitation. Future studies should explore how to directly involve children and young people as consumers to better capture their unique perspectives.

### 4.2. Implications for Practice

Pediatric healthcare needs are dynamically changing. Investment in partnering with consumers and HCPs is therefore paramount to ensure ongoing research relevance continues to improve health outcomes among the pediatric population. By defining the current research priorities for pediatric hospital care within a regional whole‐of‐life pediatric department, research efforts can be guided to ensure resources focus on areas of greatest need, translating findings into optimal benefits for consumers and the health system.

## 5. Conclusion

This study has established a shared consumer‐ and HCP‐driven research agenda within an Australian regional pediatric setting, employing a modified JLA approach. Five priority areas directly relevant to regional pediatric hospital care were established. Prioritizing child and family experience, trauma‐informed care, and adolescent and young adult health research themes demonstrates the essential role of inclusive, community‐oriented research in addressing healthcare gaps that may otherwise be overlooked in specialist or traditional tertiary pediatric settings. This approach not only highlights the feasibility of engaging a regional population in pediatric research prioritization but also sets a precedent for integrating consumer perspectives into pediatric healthcare more globally.

## Author Contributions

A.J. coordinated the study process, data acquisition, and analysis and prepared the initial manuscript draft. B.M. contributed to project design and process and reviewed and analyzed data. C.T. contributed to study conception and design, data acquisition, and analysis. J.C. contributed to study conception and design, data acquisition, analysis, and supervision. R.C. led the study conception and design, provided guidance throughout data acquisition and analysis, and facilitated the final prioritization workshop. All authors contributed to and edited the final manuscript.

## Funding

This study was funded by Wishlist (10.13039/100009725). Open access publishing facilitated by University of the Sunshine Coast, as part of the Wiley ‐ University of the Sunshine Coast agreement via the Council of Australasian University Librarians.

## Conflicts of Interest

The authors declare no conflicts of interest.

## Supporting Information

Additional supporting information can be found online in the Supporting Information section.

## Supporting information


**Supporting Information 1.** File S1 provides the full content of the PaedGROW Harvesting Survey, designed to identify future research priorities in pediatric healthcare within a regional hospital and health service. It includes tailored survey sections for both parents/caregivers and HCPs, inviting them to contribute questions and concerns based on their experiences. The materials outline the survey structure, participant eligibility, thematic focus areas (e.g., treatments, diagnostics, communication, and models of care), and demographic questions to ensure diverse representation. These responses will inform a prioritization process and subsequent workshop to guide future pediatric research initiatives.

**Supporting Information 2.** File S2 provides a visual graphic of the Top 5 research priorities.

## Data Availability

The data that support the findings of this study are available upon request from the corresponding author. The data are not publicly available due to privacy or ethical restrictions.
