# Supplementary material for: Pediatric Hospital Care Research Priorities Within a Regional Whole‐of‐Life Health Service: Established Through Consumer and Healthcare Professional Collaboration
Source: Int J Pediatr. 2026 Aug 28;2026:7312620. doi: 10.1155/ijpe/7312620 (PMC13525326; doi:10.1155/ijpe/7312620)
Supplement: Supplementary file 1 [file IJPE-2026-7312620-s001.pdf]

Our Top 5 Research Priorities for

Paediatric Hospital Care

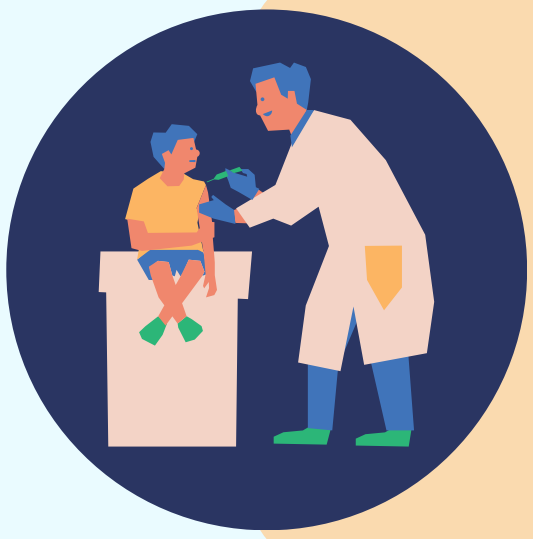

What are ways to reduce healthcare related trauma and traumatic interactions for paediatric patients and their families?

- Strategies to improve support during clinical interventions such as nasogastric insertion; diagnostic procedures; needle procedures
- Establish and evaluate psychological and physical safe spaces to access and receive paediatric healthcare

How do we better provide paediatric-centred support strategies and infrastructure for children and their families during hospital admission?

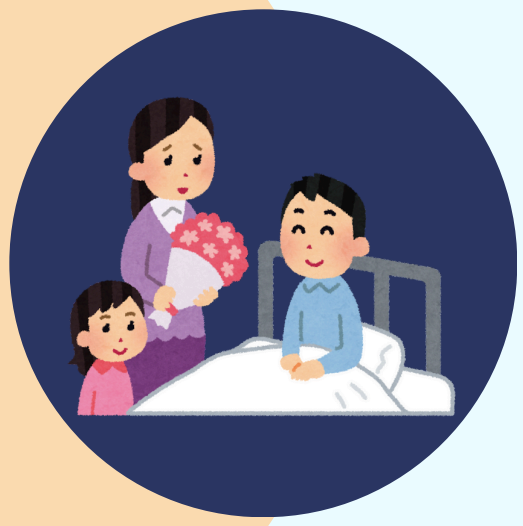

- Investigate sibling and parental needs to optimise the delivery of holistic care to children/young people and their families such as nutrition, sleep and mental health
- Improve healthcare experiences and outcomes through incorporating children and adolescent's preferences and needs

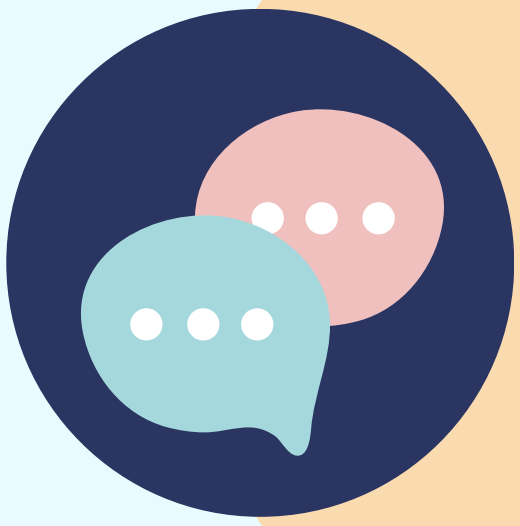

What are strategies to enhance effective communication to improve the child/adolescent and family's experience and outcomes?

- Improve consistency and clarity of the information children/adolescents and their families receive such as standardised information sharing
- Optimise continuity of care between and across treating teams
- Upscale processes for effective inter- and intra-hospital paediatric transfers

How do we enhance adolescents' and young adults' engagement, experiences and outcomes as they move from paediatric services to adult services?

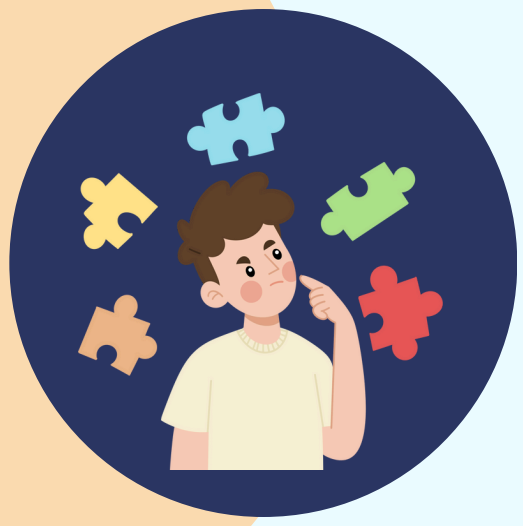

- Innovative adolescent and young adult models of care
- Address factors that build or erode trust and engagement in adolescent and young adults' healthcare

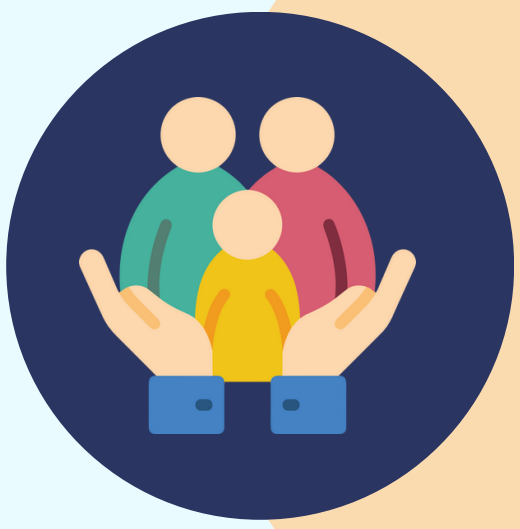

How do we improve the efficiency and quality of care as children move through a tertiary level 'whole-of-life' hospital?

- Optimise tertiary paediatric healthcare within alternative delivery settings
- Investigate facilitators and barriers to inpatient and outpatient discharge
- Enhance patient safety and upscale innovative models of care

wishlist SUNSHINE COAST HEALTH FOUNDATION
Sunshine Coast Hospital and Health Service
Queensland Government
