# Supplementary material for: Pediatric Hospital Care Research Priorities Within a Regional Whole‐of‐Life Health Service: Established Through Consumer and Healthcare Professional Collaboration
Source: Int J Pediatr. 2026 Aug 28;2026:7312620. doi: 10.1155/ijpe/7312620 (PMC13525326; doi:10.1155/ijpe/7312620)
Supplement: Supplementary file 2 [file IJPE-2026-7312620-s002.docx]

# **Supplementary File 1: Harvesting Survey**

**We would like your help to identify future research priorities for children and young persons’ health care within the [blinded] Hospital and Health Service region.**

This questionnaire will take between 5 to 10 minutes to complete.

We are a team of parents, patients, healthcare providers and researchers who are working together to identify the top research priorities related to Paediatric hospital care. By taking part in this survey, you are contributing to determining our future research project topics. This research will help us inform best practice in these areas to improve our children’s health outcomes.

If you are a:

- Parent/caregiver of a child/young person who is currently accessing care, or who has accessed care in the past 3 years.
- Health service staff (including allied health, nursing, medical and other Hospital and Health Service staff) who have provided care to a paediatric patient in the past 12 months.

**We want to hear from you!**

Think about your own personal or professional experience. What concerns, comments, or questions about health care provided to children or young people and their families would you like answered by research? **Your questions will help us direct research to find solutions and further improve paediatric healthcare.**

Think broadly about your questions, especially areas of care that could be improved. Topics may include questions about:

- Treatments, including medications (e.g. injections, pills, syrups, puffers, or ointments), diet, feeding, occupational/physiotherapy, etc.
- Tests, including blood tests, urine tests, or imaging such as x-rays, ultrasound, CT, MRI scans, etc.
- Other aspects of care, including communications, interactions with healthcare professionals, use of technology, patient safety, monitoring in hospital, etc

Some examples of research questions that have been answered include:

- Does including interpreters for all communications between healthcare professionals and families/patients, where English is not their first language, improve care for children?
- Is phototherapy the most effective treatment for hospitalised infants with jaundice?
- Does having continuity of healthcare providers improve outcomes?

All responses will be treated confidentially, and no one will know who has provided responses or suggested a particular research question or idea.

Do you agree to participate?

🞏 Yes

🞏 No

Which of the following best describes you?

🞏 Caregiver or parent of a child or young person who is currently accessing paediatric health care at [blinded] *Go to – Parent/caregiver questions*

🞏 Caregiver or parent of a child or young person who has accessed Paediatric health care at [blinded] hospital in the past 3 years *Go to – Parent/caregiver questions*

🞏 Health service staff (including allied health, nursing, medicine and other staff) who have provided care to a child or young person in the past 12 months	*Go to – Clinician Questions*

### Parent/caregiver questions

**Tell us what you think**:

Your experience and knowledge are essential in helping us identify the most important questions for future research in paediatrics. **We want to know what matters most to you.**

Think about your experience and the health care you received for your child/young person. Think about questions you may have had during their care that were left unanswered, were confusing or that you may have been curious about.

What questions do you have about paediatric healthcare that you think could be answered by research? If you can't think how to write it as a question, please tell us in your own words.

Text Box _________________________

If your child is willing and able to respond, do they have questions about their hospital care or treatments that we could improve through doing some research?

Text Box (optional response)____________

**Please tell us a little about yourself**:

We will use this information to make sure we are reaching a wide range of people to best understand the needs of different groups and experiences.

Where at the [blinded] did you/do you access health care for your child/young person?

(Please tick all that apply)

🞏 [blinded] paediatric in-patient hospital ward

🞏 [blinded] paediatric out-patient clinic

🞏 [blinded] paediatric in-patient hospital ward

🞏 [blinded] paediatric out-patient clinic

🞏 Other _______

What was your child/young person’s age at the time of last accessing healthcare?

🞏 Under 3 months

🞏 4 months to <2 years

🞏 2 to 5 years

🞏 6 to 9 years

🞏 10 to 13 years

🞏 14 to 16 years

🞏 16 and over

How many times has your child/young person been admitted to hospital as an in-patient in the last 3 years?

🞏Never *Go to – Question 7*

🞏1-2 times *Go to – Question 6*

🞏3-5 times *Go to – Question 6*

🞏More than 5 times *Go to – Question 6*

Has your child/young person accessed care through the hospital Paediatric out-patient clinic in the past 3 years?

🞏No

🞏Yes, in the last 12 months

🞏Yes, longer than 12 months ago

Does your child identify as:

🞏Female

🞏Male

🞏Prefer not to say

🞏Other (free text option)

Does your child identify as Aboriginal and/or Torres Strait Islander?

🞏Aboriginal

🞏Torres Strait Islander

🞏Aboriginal and Torres Strait Islander

🞏Neither Aboriginal nor Torres Strait Islander

Do you identify as:

🞏Female

🞏Male

🞏Prefer not to say

🞏Other (free text option)

Do you identify as Aboriginal and/or Torres Strait Islander?

🞏Aboriginal

🞏Torres Strait Islander

🞏Aboriginal and Torres Strait Islander

🞏Neither Aboriginal nor Torres Strait Islander

Please describe in a few words the main reason why your child/young person has accessed health care at the hospital. _______________________

We thank you for your time spent taking this survey, your responses have been recorded.

After we collect all the responses to this survey, we will be undertaking an interim ranking exercise to determine the top 20-30 areas. A workshop will then be held to discuss together the data and agree on future priorities for research.

If you would like to receive a link to the online ranking survey when available or to find out more about attending the workshop, please click this link to provide us with your email address. Or alternatively please contact the research team.

### Clinician Questions

**Tell us what you think:**

Think about your experience as a healthcare professional providing paediatric health care at the XX. Your experience and knowledge are essential in helping us identify the most important questions to be addressed by future research. **We want to know what matters to you.**

Think about questions you have about paediatric healthcare. Are there any questions about patient care or areas where you feel there should be more evidence to guide care?

 Please think broadly about your ideas, which may include questions about:
 ▪ **Care pathways and education:** including medications, co-morbidity management, risk assessment, clinical guidelines, etc
 ▪ **Tests & monitoring**: including pathology and imaging
 ▪ **Other aspects of care:** including communication, interactions with healthcare practitioners, use of technology, patient safety, models of care, innovation, and cultural safety

What questions do you have about paediatric healthcare that you think could be answered by research? If you can't think how to write it as a question, please tell us in your own words. Do not feel like you need to check if your question or idea has already been answered by research.

Text Box _____________

**Please tell us a little about yourself**

We will use this information to make sure we are reaching a wide range of people to best understand the needs of different groups and experiences.

What is your healthcare service role? ________

Where at the [blinded] do you provide health care services for children/young people?

(*Please tick all that apply*)

🞏 [blinded] paediatric in-patient hospital ward

🞏 [blinded] paediatric out-patient clinic

🞏 [blinded] paediatric in-patient hospital ward

🞏 [blinded] paediatric out-patient clinic

🞏 Child, Youth and Family community services

🞏 Other _______

How many years of experience do you have providing healthcare services to children and young people?

🞏 Less than 1 year

🞏 1-2 years

🞏 3-5 years

🞏 More than 5 years

Do you identify as:

🞏 Female

🞏 Male

🞏 Prefer not to say

🞏 Other (free text option)

Do you identify as Aboriginal and/or Torres Strait Islander?

🞏 Aboriginal

🞏 Torres Strait Islander

🞏 Aboriginal and Torres Strait Islander

🞏 Neither Aboriginal and/or Torres Strait Islander

We thank you for your time spent taking this survey, your responses have been recorded.

After we collect all the responses to this survey, we will be undertaking an interim ranking exercise to determine the top 20 areas, then holding a workshop for consumers and clinicians to discuss the data and agree on future priorities for research.

If you would like to receive a link to the online ranking survey when available or to find out more about attending the workshop, please click this link to provide us with your email address. Or alternatively please contact the research team.

# **Supplementary File 2: Top 5 Research Priorities**
